# Impact of Mānuka Honey on Symptoms and Quality of Life in Individuals With Functional Dyspepsia: Protocol for a Feasibility Randomized Controlled Trial

**DOI:** 10.2196/66417

**Published:** 2025-05-21

**Authors:** Laura Ombasa, Jody Miller, Lara Ware, Holly Abbotts-Holmes, Jeffry Tang, Olivier Gasser, Karl Fraser, Simone Bayer, Roslyn Kemp, Rory Costello, Andrew Highton, Jackie Evans, Troy Merry, Michael Schultz, Chris Frampton, Richard Gearry, Warren McNabb, Nicole Roy

**Affiliations:** 1 Department of Human Nutrition University of Otago Dunedin New Zealand; 2 Riddet Institute Massey University Palmerston North New Zealand; 3 High-Value Nutrition National Science Challenge Auckland New Zealand; 4 Malaghan Institute of Medical Research Wellington New Zealand; 5 AgResearch Palmerston North New Zealand; 6 Department of Medicine University of Otago Christchurch New Zealand; 7 Department of Microbiology and Immunology University of Otago Dunedin New Zealand; 8 Comvita NZ Limited Paengaroa New Zealand; 9 Department of Medicine University of Otago Dunedin New Zealand; 10 Statistecol Christchurch New Zealand

**Keywords:** functional dyspepsia, quality of life, mānuka honey, Lepteridine, randomised controlled trial, feasibility protocol

## Abstract

**Background:**

Functional dyspepsia is a common gastrointestinal condition that reduces the quality of life and increases health care costs. The lack of well-defined causes limits effective treatments. Consumers report using mānuka honey to treat gastrointestinal symptoms, although clinical evidence supporting such use is limited. Preclinical studies suggest its unique bioactive compounds may reduce gastrointestinal inflammation. Recently, 3,6,7-trimethyllumazine (Lepteridine), a natural pteridine in mānuka honey, was shown to inhibit enzymes involved in gastrointestinal inflammation in in vitro studies. Therefore, Lepteridine-standardized mānuka honey may deliver digestive health benefits.

**Objective:**

The aim of this feasibility study is to gather the data required to estimate sample size and support study logistics to design future trials. The primary objective will be preliminary assessments of the impact of Lepteridine-standardized mānuka honey on symptom severity and the quality of life in participants with mild-to-moderate functional dyspepsia. Other feasibility objectives include assessing the biological responses to mānuka honey standardized to medium and high levels of Lepteridine and measuring mānuka honey–derived metabolites in blood and urine.

**Methods:**

This is a 3-arm, parallel, controlled, double-blind, randomized feasibility study. A total of 75 healthy adults with symptoms of functional dyspepsia (Rome IV criteria) and mild-to-moderate dyspepsia severity (Short Form Leeds Dyspepsia Questionnaire) were recruited between October 2022 and September 2023. Participants were randomized into one of three groups: (1) mānuka honey standardized to contain 10 mg/kg Lepteridine, (2) mānuka honey standardized to contain 40 mg/kg Lepteridine, or (3) honey maple flavored syrup control. After a 2-week lead-in period, participants consumed 10 g of allocated intervention twice daily for 6 weeks. Throughout the study, participants completed daily bowel movement diaries and validated weekly questionnaires about their gastrointestinal symptoms and quality of life. Stool samples and 3-day diet records were collected at baseline and the end of the intervention. Blood samples were collected at baseline, weeks 2 and 4, and at the end of the intervention. In addition, 6 healthy participants without symptoms of functional dyspepsia were recruited to undergo an acute 5-hour assessment for the appearance of Lepteridine and related metabolites in plasma and urine following consumption of Lepteridine-standardized mānuka honey. The study was approved by the Northern B Health and Disability Ethics Committee.

**Results:**

Initial analysis includes 68 participants, with laboratory and data analyses being undertaken as of March 2024. The results of the primary and secondary outcomes will be published in peer-reviewed journals.

**Conclusions:**

This study will provide essential information on the potential efficacy and suitability of Lepteridine-standardized mānuka honey for designing future clinical trials investigating its effect in treating symptoms of functional dyspepsia.

**Trial Registration:**

Australian New Zealand Clinical Trials Registry (ANZCTR) ACTRN12622001140741p; https://anzctr.org.au/Trial/Registration/TrialReview.aspx?id=384094

**International Registered Report Identifier (IRRID):**

DERR1-10.2196/66417

## Introduction

### Background

Functional dyspepsia (FD) is a common gastrointestinal disorder characterized by epigastric pain, early satiety, postprandial fullness, and epigastric burning sensations that occur without explanation of the symptoms at upper gastrointestinal endoscopy [1]. The prevalence of FD ranges from 10% to 40% and varies depending on the country or criteria used to define its presence [2]. FD is more common in women than in men, which could be attributed to sex-specific differences in gastrointestinal motility caused by sex hormones, responses to pain signaling, or health-seeking behavior [3]. Currently, the pathophysiology of FD is only partially elucidated, with growing evidence implicating multifactorial causes [4]. Several pathophysiologic mechanisms have been proposed to cause FD [1,4] which include altered gastric motility [5,6], visceral hypersensitivity [7,8], altered gut microbiota composition [9-11], and gastroduodenal inflammation characterized by lymphocyte infiltration and increased eosinophils [12-14]. Psychological factors also play a crucial role in FD, as the gut and brain communicate through the enteric nervous system and hypothalamic pituitary adrenal axis [15].

FD is not life-threatening but can negatively impact health-related quality of life [16], work productivity [17], and increase health care expenses [18]. This disorder is complex and challenging to treat, primarily because of its heterogeneous nature and absence of well-defined underlying causes [19,20]. Current treatment options for FD include the eradication of *Helicobacter pylori*, use of proton pump inhibitors, H_2_ receptor antagonists, antidepressants, or prokinetic agents [19]. These existing treatments often fail to provide consistent relief, and side effects can limit their use [21]. Individuals often seek alternative therapies because of the severe impact of FD on health-related quality of life and suboptimal treatment options; however, these alternative therapies lack clinical evidence.

Honey has been an essential component of ancient medicine, with uses including preventing and treating digestive diseases [22]. Honey contains many nutrients, such as sugar, proteins, enzymes, vitamins, amino acids, and many phytochemicals [23]. The composition of honey varies depending on the flora from which nectar is collected, the type of bee that produces it, and environmental conditions. The variation in the composition of honey contributes to its diverse potential health benefits, making it useful in therapeutic therapies [24]. Studies suggest that honey could have gastroprotective properties due to its anti-inflammatory, antimicrobial, and antioxidant properties [25,26]. In addition, honey could help promote beneficial gut microbiota [27-29]. Few studies have examined the effect of honey on FD. For example, when combined with standard treatment, the adjuvant supplementation of a honey-based formulation of *Nigella sativa* significantly improved the symptoms of FD [30]. A clinical trial that examined the effects of honey and diet education on FD reported improvements in symptoms, although honey alone did not have significant gastrointestinal effects [31]. A significant improvement in symptoms was observed when honey was combined with proton pump inhibitors in children with FD [32]. However, these studies use honey as an adjuvant intervention to standard therapy, making it difficult to attribute any observed effects to honey.

Mānuka honey is a monofloral honey produced from the nectar of the *Leptospermum scoparium* tree. Mānuka honey has unique bioactive compounds, including phenolic acids, flavonoids, 1,2-dicarbonyl compounds, and pteridines [33]. Studies suggest these unique compounds [34] elicit anti-inflammatory, antioxidant, or antibacterial effects [35-37]. In vivo studies indicate mānuka honey may have gastroprotective properties by inhibiting gastric lesions and preserving gastric mucosal glycoproteins [35,36,38]. Mānuka honey also decreased the production of inflammatory cytokines such as tumor necrosis factor-alpha (TNF-α), interleukin (IL)-1β, and IL-6 [35,37]. These proinflammatory cytokines may play a key role in gastric lesions, as TNF-α is a key modulator in gastric mucosal cell death [39]. Different doses of mānuka honey provided protection against trinitrobenzene sulfonic acid–induced colitis in Wistar rats [36] and acetic acid-induced gastric lesions in Sprague-Dawley rats [35]. Furthermore, 4 weeks of oral application of mānuka honey led to changes in gut microbiota composition in mice with colorectal cancer [27]. While these studies suggest a plausible link between mānuka honey and digestive health, no clinical trials have investigated its effect on FD.

3,6,7 Trimethyl lumazine (Lepteridine) is a recently discovered pteridine derivative unique to mānuka honey [40]. Pteridines are aromatic compounds that are produced naturally by living organisms. They play essential metabolic roles, including enzymatic cofactors and immune system activation molecules. They may also have activity in the treatment of chronic inflammatory diseases [41], with methotrexate, a pteridine-derivative medicine, being used in the treatment of inflammatory bowel disease, rheumatoid arthritis, and psoriasis. Recent in vitro studies have shown that Lepteridine may contribute to the anti-inflammatory properties of mānuka honey by inhibiting the proteolytic activity of matrix metalloproteinase-9 (MMP-9) [42], which is key in maintaining the integrity of the gastric mucosa and promoting wound healing [43,44]. Lepteridine might work synergistically with other bioactive properties in mānuka honey to amplify its anti-inflammatory properties. However, the natural variation in Lepteridine concentration in mānuka honey necessitates the need for standardized formulations to ensure consistent therapeutic efficacy [42]. Currently, no clinical trials have been conducted to evaluate the potential therapeutic effects of mānuka honey standardized to Lepteridine concentration in treating FD.

### Objectives

The SOOTHE (Study of Mānuka Honey for Digestive Health) feasibility study aims to gather information required to design and undertake future clinical trials investigating the effect of Lepteridine-standardized mānuka honey on FD. The primary objective is to assess the effect of 6-week consumption of Lepteridine-standardized mānuka honey on symptom severity and quality of life in FD. This will be measured by changes in global Nepean Dyspepsia Index (NDI) scores from baseline to the end of the intervention. Data on recruitment time frame, strategies to improve recruitment, potential issues with adherence and attrition, and strategies to optimize adherence shall also be collected.

Secondary objectives include examining changes in Patient-Reported Outcome Measurement Information System (PROMIS gastrointestinal, anxiety, and depression) scores, immune parameters, and the gut microbiota composition between the Lepteridine-standardized mānuka honey and control groups. The study will also explore the pharmacokinetic profile of Lepteridine and other mānuka honey–derived metabolites in plasma and urine following acute consumption of Lepteridine-standardized mānuka honey.

## Methods

### Study Design

The protocol was developed in accordance with the SPIRIT (Standard Protocol Items: Recommendations for Intervention Trials) guidelines for clinical trial protocols [45]. Multimedia Appendix 1 provides a complete checklist of SPIRIT Outcomes 2022.

### Study Overview

**Figure 1:**
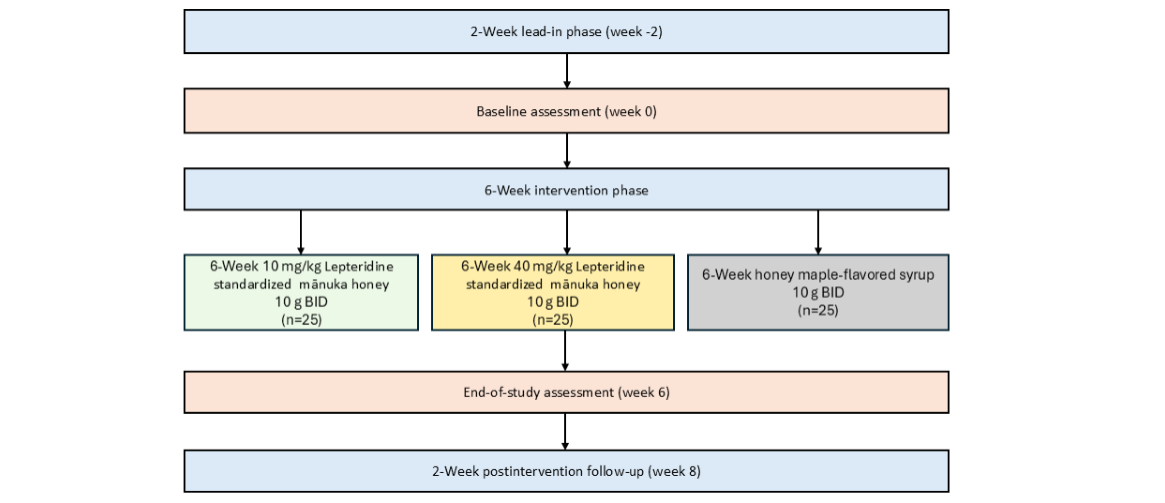
Study flowchart. BID: bis in die (10 g of allocated intervention twice daily, before breakfast and dinner).

This was a 3-arm, parallel, controlled, double-blind, randomized feasibility study in participants with FD with mild-to-moderate symptoms. The study duration was 10 weeks, consisting of a 2-week lead-in phase, a 6-week intervention phase, and a final 2-week follow-up phase (Figure 1). During the intervention phase, participants in 2 arms consumed 10 g of mānuka honey, standardized to contain either 10 mg or 40 mg/kg of Lepteridine twice daily. The third arm received 10 g of honey maple–flavored syrup twice daily as a placebo control.

Before the study commenced, a mutually agreed schedule was discussed and set with each participant to ensure they understood all expected clinic visits and the study timeline. However, participants’ needs were accommodated if they could not come on a scheduled clinic day by allowing them to attend the clinic up to 1 week later. This approach aimed to reduce the risk of participants running short of the intervention product and dropping out due to scheduling issues.

### Recruitment

Recruitment was conducted between October 2022 and December 2023. Adults from the community who self-identified as having mild-to-moderate symptoms of FD contacted the study team (through email, phone, or text) in response to study posters, radio, newspaper, or social media (Facebook and Instagram [Meta]) advertisements, word-of-mouth referrals, or from community outreach activities undertaken by the study team and a specialist clinical trial recruitment company (Trialfacts). Potential participants were then given a unique identifier link to the internet-based study information sheet and consent form. All eligible respondents were allowed sufficient time to consider participation in the study. Once informed consent was given, they completed a screening questionnaire to ascertain their eligibility for participation in the study. The screening questionnaire included the Rome IV Diagnostic Questionnaire for Functional Gastrointestinal Disorders in Adults (R4DQ) [46], the Short-Form Leeds questionnaires (SF-LDQ) [47], and general health questionnaires. Research staff reviewed the completed questionnaires, and eligible respondents were invited to a screening visit at research clinics in Dunedin or Christchurch, New Zealand.

### Eligibility Criteria

All interested respondents were assessed for eligibility based on the following inclusion and exclusion criteria (Textbox 1).

**Inclusion and exclusion criteria.**

**Inclusion criteria**

- Aged between 18 and 70 years.
- BMI between 18.5 and 40 kg/m^2^
- Met the Rome IV criteria for diagnosing functional dyspepsia.
- Had Short-Form Leeds questionnaires score <23 (to exclude people with very severe dyspepsia symptoms).
- Willing to consume 10 g of intervention twice daily, provided during the intervention period.

**Exclusion criteria**

- Unwilling to give informed consent.
- Taken antibiotics within the month before starting the study.
- Use of certain prescribed medication or recreational drugs: proton pump inhibitors, H^2^ receptor antagonists, antacids, mucosal protectants, prokinetics, antidepressant drugs if they were prescribed solely for controlling dyspepsia symptoms, anticholinergic agents, cholinergic agents, and over-the-counter herbal remedies used to treat dyspepsia symptoms (participants were asked to stop taking these for 2 weeks before the start of the study if they were to be eligible for recruitment).
- Helicobacter pylori–positive (physician-diagnosed or identified during screening) or undertaking treatment for the infection.
- Alarm features associated with significant gastrointestinal or other disorders, such as abdominal pain that wakes the participant from sleep, frequent vomiting, family history of gastrointestinal malignancies suggestive of a significant hereditary cancer syndrome, lower gastrointestinal bleeding, odynophagia, and dysphagia.
- Medical history of upper gastrointestinal surgery or other significant disorders (eg, inflammatory bowel disease and celiac disease), significant cardiorespiratory disease, diabetes mellitus, significant bleeding disorders, sleep disorders, and active psychiatric conditions (major depressive disorder and schizophrenia).
- Significant weight loss (>5% of total body weight) during the 6 months before starting the study.
- Significant dietary changes within the month before starting the study (ie, being on a controlled diet or dietary weight loss regimen).
- Intolerance or allergy to honey and bee products.
- Pregnancy or breastfeeding.
- Smokers.
- Excessive alcohol intake: >20 g of pure alcohol (2 drinks)/day on average (>21 standard drinks a week).
- Inability or unwillingness to comply with the study procedures.

### Screening

During the screening visit, research staff provided further information on the study and collected fasting blood samples for standard blood chemistry analyses (eg, glycated hemoglobin [HbA_1c_], liver and kidney function, acute inflammation, and complete blood count). Participants also provided stool samples within 24 hours after attending the clinic for an *H pylori*-antigen test. Basic blood chemistry analyses and *H pylori* antigen testing were conducted by local health clinics. Participants were excluded if either the HbA_1c_ level was above 40 mmol/mol, basic blood chemistry results were twice the upper limit, or the hemoglobin level was below 120 g/L. To reduce the risk of a false-negative *H pylori* test result, they were asked to stop taking proton pump inhibitors or bismuth preparations 2 weeks before the screening visit. Height and weight measurements were also taken to calculate BMI.

### Enrollment

Upon enrollment, participants received unique links to study questionnaires. They were also instructed to download an app to monitor daily bowel movements and compliance. The *Measurements and Outcomes* section provides more details about this.

### Study Intervention

The active intervention for this feasibility study was monofloral mānuka honey standardized to contain either 10 or 40 mg/kg of natural Lepteridine. The concentration of naturally occurring compounds does not correlate with other known compounds found in mānuka honey, such as methylglyoxal. Lepteridine in mānuka honey is derived from the nectar of the mānuka. Naturally, it varies; therefore, to produce a mānuka honey product with standardized Lepteridine concentration, testing for the presence and quantity of Lepteridine in raw honey drums was undertaken, and the resulting drums were blended to achieve the desired concentration of Lepteridine in the final mānuka honey product [40]. The composition of the intervention products is shown in Table 1. Participants received the study products in unidentifiable sachets containing 10 g of Lepteridine-standardized mānuka honey or honey maple–flavored syrup solution. Participants took the assigned intervention before breakfast and dinner for 6 weeks. They were asked to avoid consuming any other honey during the study.

**Table 1:** Composition of intervention product.

Nutrient	Mānuka honey^a^ per 100 g	Honey maple–flavored syrup^b^ per 100 g
Energy (kJ)	1370	1290
Carbohydrate (g)	80	76
Sugars (g)	78	76
Protein (g)	<1	<1
Fat (g)	<1	<1
Sodium (mg)	12	18

^a^Sourced from Comvita New Zealand.

^b^Sourced from New Zealand Sugar Company.

### Randomization, Blinding, and Allocation

A week before the baseline visit, participants were randomly assigned to a 1:1:1 allocation using randomized permuted blocks (block size 4) to either the Lepteridine mānuka honey or control groups. An independent researcher allocated participants to their intervention group using a computer-generated randomization list.

Participants and the research team were blinded to the intervention allocation. The manufacturer of the honey was responsible for labeling the intervention products to maintain the blinding of research team members. A specified research team member was responsible for enrolling participants, giving the intervention, and managing the stock. Unblinding will only be permissible once the statistical analyses of the primary outcomes are completed.

### Measurements and Their Timing

After enrollment, study clinic visits were undertaken at baseline, weeks 2, 4, and 6 (end of intervention). Currently, there is no information on the potential time course for which a treatment effect might be observed. Therefore, the multiple assessment time points enabled monitoring of potential symptom trends and identified optimal time points when any treatment effect can be observed. Table 2 shows when the study measurements were done. The information from this feasibility study will be used to determine the appropriate timing and selection of sensitive measurements for future trials.

**Table 2:** Timing of study measurements.

Measurement	Lead-in week –2	Lead-in week –1	Baseline	Week 1	Week 2	Week 3	Week 4	Week 5	Week 6	Week 7	Follow-up week 8
Nepean Dyspepsia Index	✓		✓		✓		✓		✓		✓
Anthropometry^a^			✓		✓		✓		✓		
3-Day diet record		✓							✓		
Patient-Reported Outcomes Measurement Information System	✓	✓	✓	✓	✓	✓	✓	✓	✓	✓	✓
Daily bowel diary	✓	✓	✓	✓	✓	✓	✓	✓	✓	✓	✓
Blood samples			✓		✓		✓		✓		
Stool samples			✓						✓		

^a^Measured height, weight, and waist circumference at baseline and week 6; measured weight only at weeks 2 and 4.

### Outcome Measurements

#### Primary Feasibility Measurement

The primary feasibility outcomes are changes from baseline to week 6 in FD symptoms and FD health-related quality of life, which were assessed using the NDI questionnaire. This is a validated instrument with a 2-week recall that evaluates the severity of symptoms and its impact on quality of life [48].

The symptom instrument of the NDI consists of 15 common dyspeptic symptoms that are rated using a Likert scale that evaluates the frequency, intensity, and bothersomeness of each symptom. The scores for frequency, intensity, and bothersomeness are summed to give a score for each symptom with a possible score range of 0 to 13. Higher scores represent greater symptom severity. The total symptom score is calculated as the sum of scores for all 15 individual symptoms and can range from 0 to 195. The total symptom scores are not scaled to 100, and no minimum clinically important difference (MCID) has been established [49].

The quality-of-life component of the NDI includes 25 questions that are summed to form a global score and scores for 4 subscales (or domains) measuring the impact of the condition on enjoyment of life and emotional well-being, lack of knowledge and control over the illness, disturbance to eating or drinking, and disturbance to sleep. The scores are scaled to 100 and are sometimes reversed so that high scores indicate better quality of life [50]. The quality-of-life scales can also be weighed to rank the importance of the domain to the respondent. An MCID of 10 points has been established for the NDI-weighted quality-of-life instrument [48].

In addition to changes in mean scores, responder analyses will be conducted based on the percentage of participants who demonstrated improvement in NDI scores from baseline to follow-up of 10%, 20%, 30%, and 40% for symptom severity, and 10, 20, and 30 points for quality of life. Furthermore, the percentage of participants reporting an improvement equal to or exceeding the MCID for quality of life will be compared between the groups. Effect sizes and SDs in symptom changes and quality of life between and within groups will help identify key outcome measures and sample sizes required for future studies.

Additional feasibility information was collected to inform the design of future trials. This information included response and enrollment rates and strategies, data on participant adherence to protocol, perceptions of protocol, and attrition rates. Upon exiting the study, a research team member interviewed the participants on their experiences participating in the study, using open-ended questions.

#### Secondary Measurements

The Patient-Reported Outcomes Measurement Information System (gastrointestinal, anxiety, and depression) was collected weekly. This validated system with multiple domains evaluates patient-reported outcomes that significantly impact the quality of life [51]. Measures from the gastrointestinal, anxiety, and depression domains were used as gastrointestinal disorders can impair health-related quality of life, leading to physical and mental distress [52]. The measures used evaluate gastrointestinal symptoms (bowel movements, difficulty in swallowing, belly pain, bloating, and reflux), anxiety, and depression by severity rating from “not at all” to “very much” and from “never” to “always” [51]. The data collected will provide additional information on possible beneficial or adverse effects of Lepteridine mānuka honey or placebo in individuals with FD.

A daily bowel habit diary was collected upon enrollment and throughout the study through a mobile app or paper version. The diary provided a comprehensive record of the participants’ bowel habits. It had variables on the frequency of bowel movements, which included questions on spontaneity and completeness of bowel movement, ease of defecation or level of straining, and stool form based on the Bristol Stool Scale [53].

A 3-day weighed food record was collected a week before baseline and during week 6. Participants were given an electronic dietary scale (Model 1023 WHDR 14, Salter Electronic, Salter House Wares) to weigh the food items they consumed. They also received verbal and written instructions on completing the food record, which covered 2 nonconsecutive weekdays and one weekend. The food record included written prompts for detailed information about time, place, type of food or drink, brand details, preparation or cooking methods, quantity, and a section for details on recipes. Participants were asked to record details that could have affected their usual food intake. Participants completed a paper-based version of the 3-day weighed food record during the week before their baseline and week 6 visits and returned the records during their respective visits. The food records were reviewed by a member of the research team at the time of collection, and queries were clarified with the participant. Data from the food records will be entered into a dietary analysis software, FoodWorks 10 (version 10.0, Xyris Brisbane, 2019). The New Zealand Food Composition Database (FOODfiles 2018, version 1.0, The New Zealand Institute for Plant and Food Research Limited and the New Zealand Ministry of Health) will be used to estimate energy and nutrient intakes. These data will be used to determine habitual food intake and identify dietary patterns and symptom-triggering foods. The findings will then be linked to symptom severity and gut microbiota.

### Biological Measurements

#### Blood Samples

A trained phlebotomist collected fasting peripheral blood at each visit (baseline, week 2, week 4, and week 6). Blood was collected into 10-mL EDTA tubes (Becton, Dickinson and Company) for peripheral blood mononuclear cells (PBMCs) extraction, 2 EDTA tubes (4 mL each) to measure insulin, glucose, and alpha-1 acid glycoproteins levels, and 1 serum tube without clot activators (4 mL) to measure cytokine, MMP-9, lipid, and high-sensitivity C-reactive proteins concentrations. Blood samples collected in the EDTA tubes were kept in a chilled box with ice packs until processing. Blood samples collected in the serum tubes were kept at room temperature for 45 minutes to allow coagulation before processing. Vacutainers (excluding those for PBMC extraction) were centrifuged at 2200 *g* for 10 minutes at 4 ℃, and aliquots were stored at –80 ℃ until analysis.

The PBMCs were extracted using standard procedures (Multimedia Appendix 2) at the Department of Microbiology and Immunology, University of Otago, Dunedin, New Zealand, and Mātai Hāora-Centre for Redox Biology and Medicine laboratory at the University of Otago, Christchurch, New Zealand. The extracted cells were stored in liquid nitrogen until analyses at the Malaghan Institute of Medical Research (Wellington, New Zealand). Immuno-profiling of the PBMCs, including phenotypic and functional markers, will be performed using spectral flow cytometry.

Plasma cytokines including IL-1β, IL-6, IL-8, IL-10, IL-12p70, IL-17A, IL-18, IL-23, IL-33, interferon-α2, interferon-γ, TNF-α, and monocyte chemoattractant protein-1 will be measured using a cytokine bead assay (MILLIPLEX MAP Kit Human Cytokine Chemokine Magnetic Bead Panel Immunology Assay, Millipore) as per the manufacturer’s instructions at the Malaghan Institute of Medical Research (Wellington, New Zealand).

Plasma lipids, high-sensitivity C-reactive proteins, and insulin concentrations will be measured using enzymatic assays (Abbott C series analyzer, Abbott Diagnostics), immunoturbidimetric assay (Cobas c311 analyzer, Roche Diagnostics), and Cobas e411 analyzer (Roche Diagnostics) respectively, at the Canterbury Health Laboratories. The remaining plasma and serum samples will be analyzed at the Department of Human Nutrition, University of Otago, Dunedin Campus. For this, serum MMP-9 concentration will be measured using enzyme-linked immunosorbent assay (Human ELISA Kit, Invitrogen, Thermo Fisher Scientific), plasma glucose will be measured by enzymatic assays (Abbott C Series analyzer, Abbott Diagnostics), and alpha-1 acid glycoproteins will be measured using immunoturbidimetric assay (Cobas c311 analyzer, Roche Diagnostics).

#### Stool Samples

Once enrolled, participants submitted a stool sample using the provided OMNIgene.GUT|OM-200 (DNA Genotek) stool kit 24 hours before the clinic day at baseline and week 6. The samples were stored at room temperature until processing was done within a week. A total of 2 aliquots (2 g each) of stool samples were stored at –80°C until analysis.

DNA was extracted from the stool samples using the Macherey-Nagel NucleoSpin DNA stool kit per the manufacturer’s instructions. Only data relevant to analyzing the gut microbiome composition were extracted. Long-read Oxford Nanopore sequencing will be used for full-length 16S rRNA gene sequencing (~1500 bp) [54] to capture information from all 9 hypervariable 16S regions. Nanopore-generated sequence reads, which are re-base-called using “Super high accuracy base-calling” post run, will be passed through FastQC and then the standard EPI2ME amplicon workflow using the SILVA-138 database for taxonomic assignment.

#### Lepteridine Metabolites Acute Study

As a preliminary investigation into the postprandial absorption and excretion of selected mānuka honey-derived compounds, a randomized, crossover, acute study involving 6 healthy participants without symptoms of FD was conducted. Each participant attended 2 clinic visits, where they consumed 20 g of either of the Lepteridine-standardized mānuka honey formulations (containing 10 mg or 40 mg Lepteridine). After a 1-week washout, participants consumed the other formulation. The sequence in which they received the honey was randomly selected.

Blood and urine samples were collected before and after the participants consumed the assigned honey. To ensure they were hydrated, participants were asked to drink 250 mL of water every hour. Participants could not eat or drink any other food during the clinic except for the allocated study honey and water. Blood and urine samples were collected over 5 hours. A cannula was fixed by medically trained personnel to allow multiple blood sample collection at baseline (fasting samples) and at 0.5, 1, 2, and 5 hours after consuming the assigned honey. At each time point, 4 mL of blood was collected in a heparin and EDTA vacutainer. The blood was centrifuged at 3000 *g* for 10 minutes at 4 ℃, and aliquots were stored at –80 ℃. All urine voided during the 5-hour clinic was collected into sterile containers and stored at –80 ℃.

The analytical methods for measuring mānuka honey–derived compounds and metabolites will be based on a previous study that investigated the in vivo absorption and metabolism of Leptosperin over 3 hours [55], with amendments as required. Briefly, acidified urine will be extracted using solid-phase extraction (Strata-C18E cartridge, 3 mL, 500 mg, Phenomenex), which will be preconditioned with methanol and 0.1% acetic acid in water. After washing with 2 mL 0.1% acetic acid in water, metabolites will be eluted with 2 mL methanol and then analyzed by liquid chromatography with tandem mass spectrometry. For plasma, 20 µL of the sample will be extracted with 300 µL methanol, and the extract will be evaporated and reconstituted in 0.05% formic acid for liquid chromatography with tandem mass spectrometry. The analyses will be performed on an EXION high-performance liquid chromatography system fitted with a C18 column (Waters 1.7 µm ACQUITY UPLC BEH C18, 100 × 2.1 mm ID) attached to a SCIEX 6500+ Q-TRAP (AB SCIEX). Urine samples will be analyzed in precursor ion mode for m/z 211 (methyl syringate ion) while the plasma will be analyzed in multiple reaction monitoring modes. Biotransformer 3.0 [56] will be used to predict possible metabolites to extend the list of metabolites provided in Ishisaka et al [55]. The metabolites will be quantified using commercial standards where available.

### Data Analysis

#### Sample Size

The determinant of the sample size for this study was to enable the estimation of the SD and the potential effect size for the primary outcome. This estimation is crucial as it allows for sample size calculation in future trials, ensuring the study’s findings are robust and applicable. A sample size of 75 gives an estimated degree of freedom to calculate SDs, allowing reasonable precision.

#### Data Management

The study will generate quantitative and qualitative data, including questionnaires, laboratory data, clinically relevant information regarding symptoms, and face-to-face interviews on participants’ perceptions of the study protocol. Only data relevant to answering the research question will be collected. There was no anticipated harm during data collection beyond standard care and everyday life risks.

The data collected were in the form of electronic files, Microsoft Word documents, and a small number of paper-based questionnaires. Study data were collected and managed using the REDCap (Research Electronic Data Capture, Vanderbilt University) tool hosted at the University of Otago, Dunedin Campus. REDCap is a secure, web-based software platform designed to support data capture for research studies, providing (1) an intuitive interface for validated data capture, (2) audit trails for tracking data manipulation and export procedures, (3) automated export procedures for seamless data downloads to common statistical packages, and (4) procedures for data integration and interoperability with external sources [57,58]. It was only accessible to the researchers to contact participants for planned data collection. All participants were assigned a unique study identification number at the time of providing consent, which was recorded in REDCap and used instead of personal identifying information in all other data files. A formal data monitoring committee was not required as the intervention and control treatments in the study were considered sufficiently minimal risk. No harm was anticipated beyond the risks of standard care and everyday life. Data monitoring occurred through weekly meetings of the research team.

#### Data Collection and Storage

The researchers collecting data were trained in data collection and maintaining security and health-related confidentiality. Most of the data were collected electronically through the REDCap data capture system using unique identification. Participants who preferred paper-based data collection were accommodated. Paper-based and telephone-collected data were entered directly into the REDCap.

The data files were stored in Microsoft Excel for raw data and as data analysis files in R (version 4.3.2, R Foundation for Statistical Computing), Stata (version 18.0, StataCorp), or SPSS (version 29, IBM Corp). These data were only identified by the unique identification of the participant. Participants were identified by researchers only by their unique identification.

Raw data collected in hard copy were stored after electronic data entry as part of the case report form in a locked filing cabinet in the principal investigator’s (JM’s) office. Electronic data files generated in the study were stored on a password-secured University of Otago server or Otago OneDrive cloud storage. These files will be accessed as needed on password-protected computers of named investigators.

#### Statistical Analyses

All statistical analyses will be done using either R (version 4.3.2), Stata/SE version 18.0, or IBM SPSS version 29. The primary outcome analyses will use the intention-to-treat population (ITT) and per-protocol population. Secondary and exploratory analyses will be conducted with the ITT population. The ITT population will include all randomized participants who took at least 1 dose of the intervention. The per-protocol population will include those in ITT who had no major protocol violations, had all the study assessments required to analyze any outcome, and consumed at least 80% of their intervention product. Participants with missing data for a relevant outcome assessment will be imputed using the last observation carried forward.

The primary analysis will compare the overall NDI score changes for symptom severity and quality of life from baseline to week 6. Quality of life scores weighted by the participants’ perception of importance and unweighted scores will be examined. A general linear model that includes the randomized group as a fixed factor will be used (unadjusted results), and models adjusting for baseline values will also be presented. The residual plots from these analyses will be visually inspected, and if reasonable normality is not apparent, alternative generalized linear models will be undertaken.

For the secondary outcomes, changes in scores from baseline to the end of the intervention will be analyzed using appropriate statistical tests, depending on the data distribution. Descriptive statistics will be presented as frequencies, percentages, and mean (SD) for normally distributed parameters or medians (IQRs) for non-normally distributed parameters with a 95% CI and *P*≤.05 value to show statistical significance. Appropriate statistical techniques shall be used to mitigate any potential confounders and shall be reported in subsequent publications of this study. No formal hypothesis testing will be conducted on these variables.

Stool microbiome data will be analyzed using principal coordinate analysis and permutational multivariate ANOVA. Alpha-diversity and beta-diversity indices will be calculated. Differential abundance analysis of the microbiome abundance data (Analysis of Composition of Microbiomes with Bias Correction) [59] will be used to explore relationships between diet, metadata, diversity indices, and microbiome taxonomic traits. All analyses will be done in R with probability values adjusted for multiple comparisons using the Benjamini-Hochberg method [60] and data will be reported according to the reporting guidelines for human microbiome research (STORMS [Strengthening The Organizing and Reporting of Microbiome Studies]) [61].

### Ethical Considerations

The study was conducted in accordance with the International Conference of Harmonisation guidelines, national and local requirements, and the ethical principles originating in the Declaration of Helsinki. Ethical approval was granted by the Northern B Health and Disability Ethics Committee (reference 20222 FULL 12734). Before commencement, the study was registered on the Australian New Zealand Clinical Trials Registry (ACTRN12622001140741p). All participants were required to date and sign an informed consent before participating in the study.

## Results

Enrollment into the study began in December 2022 and was completed in September 2023. The intervention phase and data collection for the main study were completed in December 2023. A total of 75 participants were enrolled in the study. Out of 75, 7 participants withdrew before receiving the allocated intervention and were not included in the analysis, resulting in a final population of 68 participants (52/68, 76% female and 16/68, 24% male). As of March 2024, data analysis of the PROs and laboratory analyses is being undertaken, with full results expected to be published in 2025. The study’s results will be disseminated in scientific manuscripts published in peer-reviewed journals and through abstracts, posters, and presentations at national and international conferences.

## Discussion

### Anticipated Findings

FD is a common chronic gastrointestinal condition that can significantly affect the quality of life [16]. However, despite high prevalence rates, its pathophysiology is poorly understood [1,4], limiting the development of effective treatment options [21]. These factors may lead people to seek alternative therapies, although the evidence supporting the effectiveness of such therapies is limited and inconclusive [19]. Honey has been used to treat digestive diseases, based primarily on anecdotal or preclinical research [22,30,38]. Emerging evidence suggests honey might promote healthy gut microbiota composition [27,28,62]. Mānuka honey has many unique bioactive compounds that might lend it to be an alternative treatment for FD. Although preclinical studies have shown potential gastroprotective properties of mānuka honey, and recent in vitro evidence suggests the naturally occurring mānuka compound Lepteridine may modulate digestive inflammatory pathways, clinical studies are needed to explore and validate these findings. Recently, a pilot study reported a potential benefit of mānuka honey on symptoms of gastroesophageal reflux disease [63]. However, well-designed clinical studies have yet to test these postulated benefits.

The SOOTHE study is a randomized, controlled, feasibility study designed to gather crucial information to inform the design and implementation of clinical trials to test the effects of Lepteridine-standardized mānuka honey on FD symptoms and the quality of life. The study involves a well-defined population group, identified using a validated diagnostic tool (Rome IV), and provides information on symptom severity using the validated SF-LDQ. In addition, using an intervention with a standardized formulation allows for consistent dosing.

Given the lack of previous clinical studies to inform this study design, we were unable to power this study to detect a significant treatment effect; however, this study will provide information as to whether further investigation into mānuka honey for FD treatment is warranted. If a treatment effect is indicated, the information gathered will allow future studies to be well-designed and adequately powered. Frequent assessments during the study period will allow for the investigation of the time course of any potential treatment effects and will help refine future study duration and study measurements.

The inclusion of 2 different formulations of Lepteridine will provide an initial indication of a potential causal effect of Lepteridine on gastrointestinal health. The inclusion of biological assessments along with patient-reported outcomes will contribute to the emerging knowledge about this poorly understood condition and can be used to support the interpretation of patient-reported results.

### Disclaimer

#### Consent for Publication

Data derived from this study will be the exclusive property of the principal investigators. No use or transmission to a third party will be made possible without their consent. The principal investigators will approve any publication or presentation related to the study. In addition, biological samples and participant data collected during this study may not be used by site investigators for research unrelated to this protocol without the previous approval of the principal investigators. Other legal aspects, including intellectual property, are covered by contracts between the High-Value Nutrition National Science Challenge-Ko Ngā Kai Whai Painga, University of Otago, Comvita New Zealand, Massey University, and Malaghan Institute of Medical Research.

#### Protocol Amendment

Any protocol changes that may affect the conduct of the study and the safety of participants, including changes in study objectives, study design, study population, and sample size, require a formal amendment to the protocol. There were amendments to the cut-off criteria for BMI from 18.5-35 kg/m^2^ to 18.5-40 kg/m^2^, allowing a wider scope of persons to be recruited into the study. In addition, adjustments were made to the design of the Lepteridine acute study. Initially, the acute study was to be done during the baseline visit in a subgroup of 5 participants in each honey group. However, the design was adjusted to a crossover design with a 1-week washout period as there is no information on the absorption and metabolism of Lepteridine honey in healthy people. However, these changes were not considered significant, although the ethics committee was informed of the changes.

## Appendix

Multimedia Appendix 1 Revised SPIRIT (Standard Protocol Items: Recommendations for Interventional Trials) checklist.

Multimedia Appendix 2 Isolation of peripheral blood mononuclear cells.

## Abbreviations

FD: functional dyspepsia
IL: interleukin
ITT: intention-to-treat
MCID: minimum clinically important difference
MMP-9: matrix metalloproteinase-9
NDI: Nepean Dyspepsia Index
PBMC: peripheral blood mononuclear cell
PROMIS: Patient-Reported Outcome Measurement Information System
R4DQ: Rome IV Diagnostic Questionnaire for Functional Gastrointestinal Disorders in Adults
REDCap: Research Electronic Data Capture
rRNA: ribosomal RNA
SF-LDQ: Short Form Leeds Dyspepsia Questionnaire
SOOTHE: Study of Mānuka Honey for Digestive Health
SPIRIT: Standard Protocol Items: Recommendations for Interventional Trials
STORMS: Strengthening The Organizing and Reporting of Microbiome Studies
TNF-α: tumor necrosis factor-alpha
