# Supplementary material for: Impact of Mānuka Honey on Symptoms and Quality of Life in Individuals With Functional Dyspepsia: Protocol for a Feasibility Randomized Controlled Trial
Source: JMIR Res Protoc. 2025 May 21;14:e66417. doi: 10.2196/66417 (PMC12138293; doi:10.2196/66417)
Supplement: Multimedia Appendix 2 [file resprot_v14i1e66417_app2.docx]

Isolation of peripheral blood mononuclear cells (PBMC):

1. The blood collected in the 3 × 10-mL vacutainers was poured evenly into 2-3 sterile 50-mL centrifuge tubes.
2. Blood volume was matched (up to 30 mL maximum) with PBS.
3. Lymphoprep (15 mL) was added into the bottom section of each of the SepMate-50 tubes.
4. The diluted blood from each of the tubes was gently pipetted down the side of each of the prepared SepMate-50 tubes and centrifuged at 1,200 g (RT, 10 minutes, acceleration: 9, deceleration: 9) on a swing-bucket centrifuge to separate the various blood components.
5. The top layer was then poured into sterile 50-mL centrifuge tubes, avoiding red blood cell contamination, topped up to 50 mL with PBS and centrifuged at 300 g (RT, 8 minutes, acceleration: 9, deceleration: 9).
6. The supernatant was discarded to remove the plasma and residual Lymphoprep.
7. Pelleted cells were pooled and resuspended in 10 mL PBS, and 10 µL was taken for cell counting.
8. The tube was centrifuged at 300 g, and the supernatant was discarded.
9. The PBMC pellet was resuspended in the freezing mixture (90% HI-FBS:10% [v/v] DMSO) at a concentration of approximately 1×10^7^ cells/mL and aliquoted into prelabeled cryovials.
10. The cryovials were transferred into a CoolCell LX freezer container and kept at −80°C overnight to slowly freeze the cells before transfer into a liquid nitrogen dewar.
